# Emergency laparoscopic ileo-colic resection and primary intracorporeal anastomosis for Crohn’s acute ileitis with free perforation and faecal peritonitis: first ever reported laparoscopic treatment

**DOI:** 10.1186/s40064-015-1619-x

**Published:** 2016-01-06

**Authors:** A. Birindelli, G. Tugnoli, D. Beghelli, A. Siciliani, A. Biscardi, C. Bertarelli, S. Selleri, R. Lombardi, S. Di Saverio

**Affiliations:** Maggiore Hospital Regional Emergency Surgery and Trauma Center–Bologna Local Health District, Bologna, Italy; Maggiore Hospital Pathology Department–Bologna Local Health District, Bologna, Italy

**Keywords:** Emergency Laparoscopy, Colorectal surgery, Intracorporeal anastomosis, Faecal peritonitis, Small bowel perforation, Crohn’s disease

## Abstract

**Introduction:**

Laparoscopy for abdominal surgical emergencies is gaining increasing acceptance given the spreading of advanced laparoscopic skills among modern surgeons, as it may allow at the same time an accurate diagnosis and appropriate treatment of acute abdomen. The use of the laparoscopic approach also in case of diffuse peritonitis is now becoming accepted provided hemodynamic stability, despite the common belief in the past decades that such severe condition represented an indication for conversion to open surgery or an immediate contraindication to continue laparoscopy. Crohn’s Disease (CD) is a rare cause of acute abdomen and peritonitis, only a few cases of CD acute perforations are reported in the published literature; these cases have always been approached and treated by open laparotomy.

**Case description:**

We report on a case of a faecal peritonitis due to an acute perforation caused by a terminal ileitis in an undiagnosed CD. The patient underwent diagnostic laparoscopy followed by a laparoscopic ileo-colic resection and primary intracorporeal anastomosis, with a successful postoperative outcome.

**Conclusions:**

Complicated CD has to be considered within the possible causes of small bowel non-traumatic perforation. Emergency laparoscopy with resection and primary intra-corporeal anastomosis can be feasible and may be a safe and effective minimally invasive alternative to open surgery even in case of faecal peritonitis, in selected stable patients and in presence of appropriate laparoscopic colorectal surgical skills and experience. To the best of our knowledge the present experience is the first ever reported case managed with a totally laparoscopic extended ileocecal resection with intracorporeal anastomosis in case of acutely perforated CD and diffuse peritonitis.

**Electronic supplementary material:**

The online version of this article (doi:10.1186/s40064-015-1619-x) contains supplementary material, which is available to authorized users.

## Introduction

Laparoscopy is nowadays widely accepted as the preferred surgical approach in most elective surgical cases because of its clear post-operative advantages when compared to open surgery. However, its role in emergency surgery is still a matter of debate. Acute abdomen is the most common finding in emergency surgery departments and it can be caused by several different diseases. This is the reason why in these patients, laparoscopy can play an important role in defining the correct diagnosis and in some cases may avoid unnecessary or non-therapeutic laparotomies, and may even allow, depending on the surgeon’s skills and experience, to perform the surgical appropriate definitive treatment. Moreover, the use of laparoscopy as a diagnostic tool can be important as an alternative to non-invasive diagnostic tools, which are expensive and not everywhere available (Sauerland et al. 2006; Branicki 2002; Di Saverio 2014; Cueto 1997; Agresta et al. 2006; Kirshtein 2003; Agresta et al. 2004; Sangrasi et al. 2013; Sinha et al. 2005). Last but not least, diagnostic tools like plain x-rays and ultrasonography are affected by poor diagnostic sensitivity and specificity, whereas CT scan, although having higher diagnostic accuracy, does not reach 100 % sensitivity and specificity and is associated to a significant radiation exposure and potential long-term morbidity (Chatu et al. 2013).

Historically, the presence of faecal peritonitis has been considered as a contraindication to laparoscopy, because of the theoretical risk of malignant hypercapnia, due to an increased absorption of carbon dioxide in the presence of severe intra-abdominal infection and inflammation of the peritoneum, and, secondly, because of the risk of toxic shock syndrome by increased passage of toxins and bacteria into the circulation favoured by the high intraperitoneal pressure. Recently, this controversial issue has been further investigated and the benefits of laparoscopy have been demonstrated also in case of peritonitis (Uzunkoy et al. 2012; Pitombo 2008; Montalto et al. 2012; Metzelder et al. 2008; Hsieh et al. 2011; Horattas et al. 2003; Hanly et al. 2003; Casaroli et al. 2011; Barbaros et al. 2004).

Crohn’s disease (CD) is an idiopathic chronic transmural inflammatory disease; it can affect the whole gastrointestinal tract from mouth to anus, even if the most common diseased site is the distal ileum. The pathological features include strictures, abscesses and fistulas. Laparoscopy is already commonly used in elective setting for well-selected uncomplicated CD surgeries (creation of stomas and limited segmental involvement bowel stenosis), but more complex patients are treated laparoscopically only in highly experienced centers (Navez et al. 1998; Stocchi et al. 2008; Msika et al. 2001; Maggiori and Panis 2014; Lee and Fleming 2012; Kessler et al. 2011; Huilgol 2004; Bergamaschi et al. 2009; Aarons 2013; Rosman et al. 2005; Duepree and Senagore 2002; Casillas and Delaney 2005; Milson and Hammerhofer 2001; Lim et al. 2014; Lesperance et al. 2009; Tilney and Constantinides 2006; Maartense et al. 2006; Patel et al. 2013; Nguyen et al. 2009; Neumann et al. 2013; Lowney et al. 2006; Goyer et al. 2009; El-Gazzaz et al. 2010a; Dunker and Stiggelbout 1998; Tan and Tjandra 2007; Dasari et al. 2011). CD free perforation in the peritoneal cavity is rare (Ikeuchi and Yamamura 2002) and the use of emergency laparoscopy in perforated CD is still largely limited by lack of specific experience in laparoscopic treatment of abdominal emergencies.

We report on a case of acute intestinal perforation with faecal peritonitis resulting from a terminal ileitis, that led to a first diagnosis of CD and that was treated with laparoscopic ileo-colic resection and primary intracorporeal anastomosis with a good outcome.

## Case report

A 61-year-old man was admitted to the emergency department after sudden onset of acute abdominal pain in the early morning.

Past medical history was positive for a previous laparoscopic cholecystectomy (September 2014), followed by chronic diarrhea treated empirically with pancreatic enzymes.

**Fig. 1:**
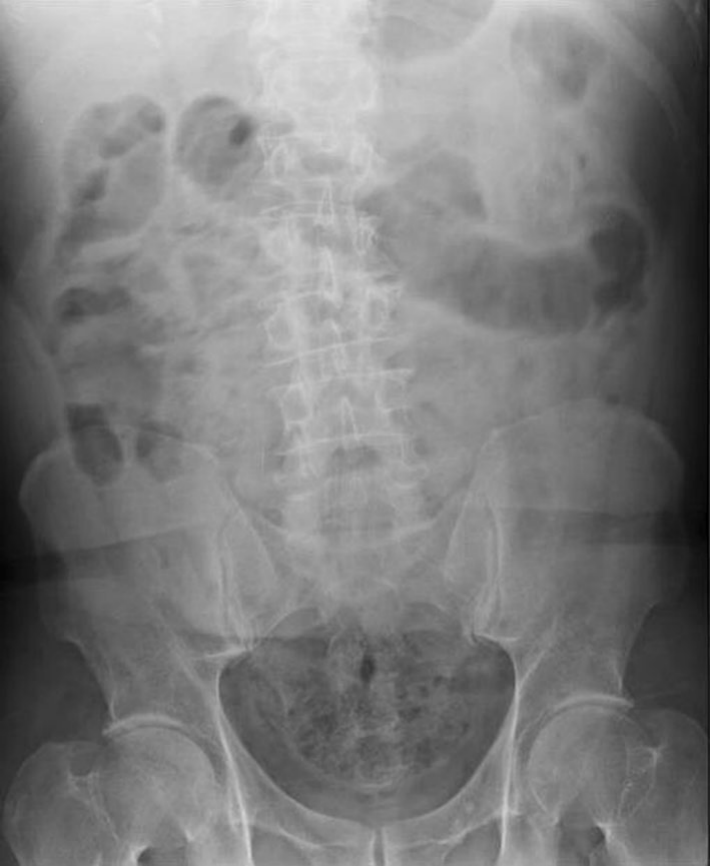
Abdominal X-ray showing diffuse coprostasis and small bowel distension, with no abdominal free air

**Fig. 2:**
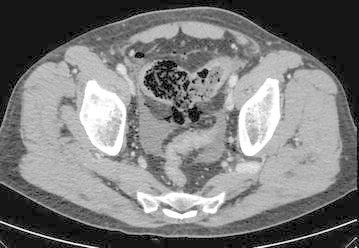
Abdominal CT-scan showing endoperitoneal free air, some thickened ileal loops and pelvic free fluids with a large pelvic collection..

Admission laboratory findings showed an elevated leukocyte count (15.330/mmc) with neutrophilia (85 %). Abdominal X-ray scan found diffuse coprostasis and small bowel distension, with air fluid levels but no abdominal free air (Fig. 1). Abdominal ultrasound reported some small bowel loops thickened and some perihepatic fluid. Computed tomography showed endoperitoneal free air, confirmed some thickened ileal loops, moderate periepatic and pelvic free fluids with a large pelvic collection and stomach dilatation (Fig. 2).

According to these findings, along with diffuse abdominal tenderness, an emergency diagnostic laparoscopy, suspecting a perforated diverticulitis, was performed. Open Hasson access was inserted in the umbilicus, followed by the positioning of two operative trocars in the right abdomen. A severe diffuse faecal peritonitis was found. After thorough and careful suction of the free fluid from all quadrants, the stomach and colon appeared to be macroscopically normal and without signs of perforation. However, the small bowel appeared extremely dilated. After changing the laparoscopic view from the left to the right quadrants of the abdomen and inserting two more operative trocars in the left abdomen, a challenging gentle mobilization of the bowel loops allowed to reach the ileum where a large semi-circumferential ileal perforation with active spillage of enteric fluid was found. Moreover, this part of the ileum appeared extremely inflamed, thickened, oedematous and hyperaemic, with associated creeping mesenteric fat and mesenteric lymphadenopathy, consisting with the suspicion of IBD.

According to the extent of the disease, we have decided to proceed to an extensive entirely laparoscopic ileal-colic resection (about 80 cm of terminal ileum and part of the ascending colon) which revealed to be technically challenging and was performed by an experienced operator with advanced laparoscopic colorectal skills (SDS). After the large bowel section performed using an endostapler device and carried out at the passage between cecum and ascending colon, the mesocolon, ileocolic vessels and mesentery of the distal ileum were sealed and sectioned intracorporeally using harmonic scalpel (Ultracision ACE 7^®^). Therefore, after appropriate medial mobilization of the cecum and distal ileum from the right flank and right iliac fossa with incision of lateral Toldt's fascia, the specimen was extracted using a wound protector, from an umbilical mini-incision, starting by pulling out the resected bowel from the stapled colonic end. after appropriate mobilization from the right flank and right iliac fossa. The vascular section of the remaining proximal small bowel was completed extracorporeally because of the intense inflammation of the small bowel mesentery and the subsequent risk of vascular damage or bleeding from the mesentery. An isoperistaltic latero-lateral intracorporeal stapled anastomosis was performed between the proximal ileum and the proximal transverse colon with manual intracorporeal closure of the enterotomy. Two J-P suction drains were positioned, one in the right hypocondrium and the other in the pelvis (see Additional file 1).

After surgery the patient was transferred to the ICU. The post-operative course was complicated by severe septic shock treated with resuscitating therapy and wide range antibiotics. Blood cultures revealed a *E. Coli*, *K. Pneumoniae* and *C. Albicans* infection. Temporary tracheostomy was needed. Early enteral feeding (via naso-jejunal tube) and passage of stools were achieved (post-operative day POD#4). At the follow-up CT performed on POD#9 because of the persistent septic shock, two intra-abdominal pelvic abscesses were found, one in the right iliac fossa and the other inter-loop in left iliac fossa. No anastomotic leaks occurred. The right abscess was evacuated, with active aspiration from the lower drain positioned on the day of surgery. An ultrasound-guided drainage of the left abscess was attempted without success and the collection was spontaneously gradually reabsorbed. A minor skin infection in the umbilical mini-incision occurred. A complete thrombosis of the right subclavian, axillar, umeral, basilica and cephalic veins also occurred and was treated with LMWH.

**Fig. 3:**
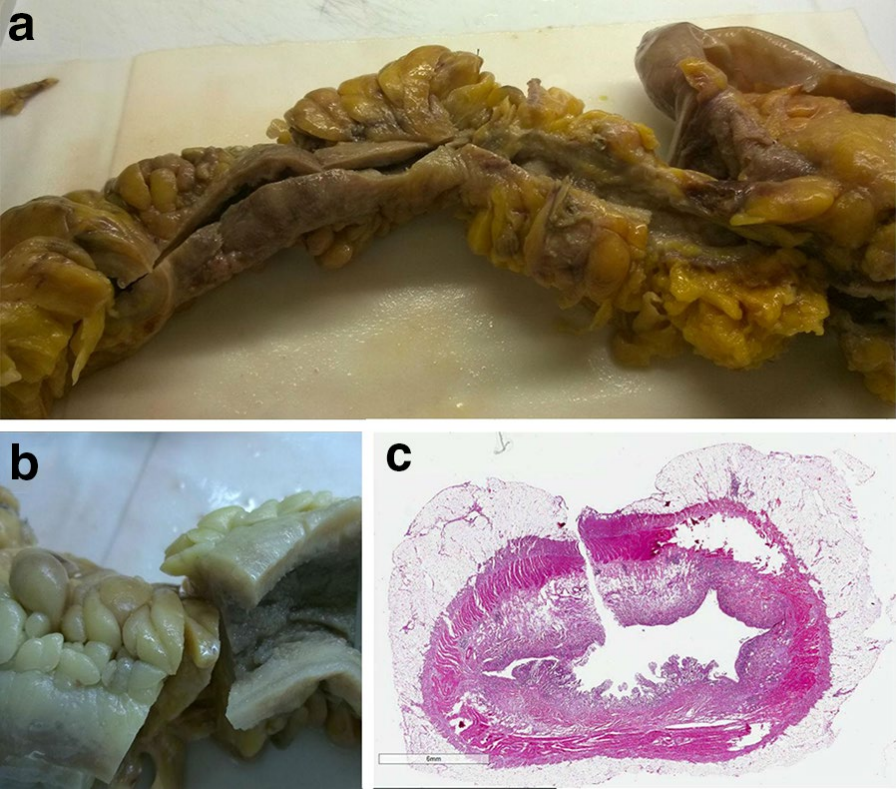
Surgical resection specimen pathology assessment. (a) (b) Gross examination of the surgical resection specimen showing Crohn's Disease macroscopic features: fibrotic and stenotic small bowel with creeping mesenteric fat, thickened wall and mucosal ulcerations and fissurations; (c) Histopathologic examination of the surgical resection specimen showing Crohn's Disease microscopic  features with deep inflammatory infiltrate, mucosal ulcerations and multiple lymphoid aggregates

The patient was discharged from the ICU on POD# 26 and from the surgical ward on day 32, after the confirmation of CD by histology (Fig. 3). Mesalazine therapy was started. Because of the long immobility of the patient, a full rehabilitation program was completed.

**Fig. 4:**
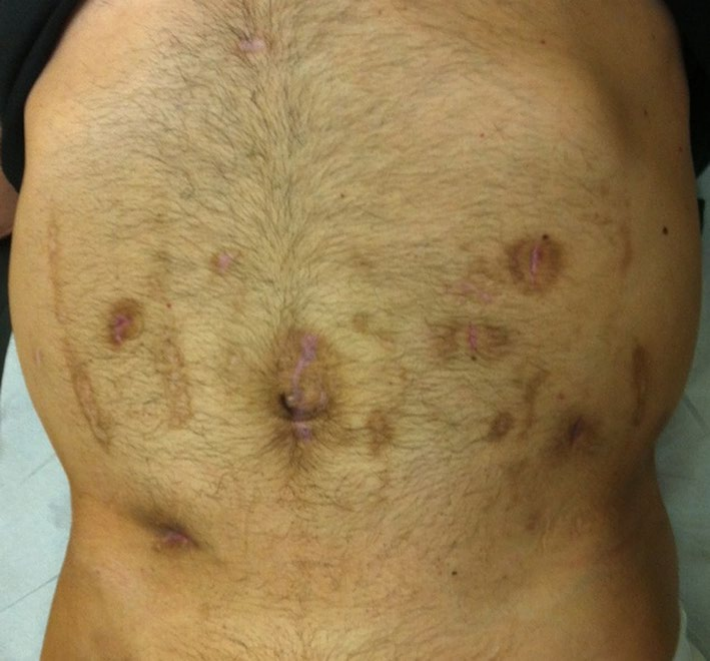
Cosmetic post-operative outcome

At gastroenterology follow-up (3 and 6 months from surgery) the patient still reported some diarrhea and haematochezia, but is in good general conditions, the blood exams are within normal range, abdomen is soft and the weight is stable (Fig. 4). Abdominal US and colonoscopy with biopsies were planned in order to diagnose for either further recurrence or other localizations of CD.

## Discussion

According to the increasing technical skills of laparoscopic surgeons, the choice of laparoscopy in emergency surgery is gaining wider acceptance, especially because it can help accomplishing the diagnosis and in most cases also the treatment of acute abdomen. In fact, unlike elective surgery where the diagnosis is usually known before the operation, in abdominal emergencies the cause of an acute abdomen is not always clearly evident. Laparoscopy may allow to explore the entire abdomen, to make a definitive diagnosis and to decide the most adequate treatment. In addition it allows a shorter surgical incision or even to avoid a large un-necessary laparotomy, with obvious post-operative benefits for the patient (Sauerland et al. 2006; Branicki 2002; Di Saverio 2014; Cueto 1997). The international literature reports a very high diagnostic accuracy of laparoscopy (85–100 %) (Sauerland et al. 2006), and in cases of unclear preoperative diagnosis, laparoscopy can shorten the observation period and avoid the need for expensive laboratory and imaging test (Cueto 1997; Agresta et al. 2006; Kirshtein 2003).

Although peritonitis used to be commonly considered a contraindication to laparoscopy, because of the theoretical concern that the CO2 pneumoperitoneum that may enhance bacteraemia and endotoxemia due to the increased intraperitoneal pressure (Nordentoft et al. 2000), the latest guidelines by EAES and most of the clinical and experimental studies support the concept that laparoscopy and minimally invasive surgery are able to produce a less inflammatory response with a less risk of kidney and lung failure, less trauma and tissue damage than open surgery (Uzunkoy et al. 2012; Pitombo 2008; Montalto et al. 2012; Metzelder et al. 2008; Hsieh et al. 2011; Horattas et al. 2003; Hanly et al. 2003; Casaroli et al. 2011; Barbaros et al. 2004; Kesici et al. 2011; Collet e Silva et al. 2000; Neudecker et al. 2002). In fact, over the past few years there has been an increasing number of studies on the use of laparoscopy in the treatment of peritonitis reporting favourable results (Sauerland et al. 2006; Branicki 2002; Di Saverio 2014; Cueto 1997; Agresta et al. 2006; Kirshtein 2003; Agresta et al. 2004; Sangrasi et al. 2013; Agresta et al. 2012).

Another common concern about the laparoscopic treatment of diffuse peritonitis is the efficacy and safety of the peritoneal lavage and toilette. However, from the first introduction of the technique better known as Laparoscopic Lavage more than 15 years ago (O’Sullivan et al. 1996), several experiences in the literature confirmed its efficacy in achieving an effective wash out of diffuse peritonitis, even with better results than open surgery if performed by experienced operator (Favuzza et al. 2009; Karoui et al. 2009), since it allows the surgeons to reach the most hidden and deep spaces of the abdominal quadrants (Myers et al. 2008).

The post-operative advantages of laparoscopy are commonly accepted. In fact, it may decrease pain, morbidity (pneumonia and wound infections) and mortality; in addition, it may increase prompt recovery of gastrointestinal functions, shorten hospital stay, carry a lower incidence of incisional hernias and lesser adhesions, thus decreasing health-care costs and allowing higher comfort and better cosmesis. All these advantages are also demostrated in emergency surgery (Di Saverio 2014; Agresta et al. 2004; Sangrasi et al. 2013; Sinha et al. 2005).The only data remaining controversial about the use of laparoscopy in acute care surgery are the possible disadvantages of longer operative times and of high intra-abdominal pressure, which both seem to have negative effects (Agresta et al. 2004, 2006). The latter can be reduced if the intraperitoneal pressure is maintained on or below 12 mmHg.

An ulcerative ileitis “Crohn-like”, as a result of the chronic pancreatic enzymes taking, has been described in the literature (Thomson and Tam 1994; Croft et al. 1995; Smyth et al. 1994; FitzSimmons et al. 1997). According to the patient's medical history, our pathologist suggested it as a possible differential diagnosis, since in the specimen, apart from strictures and submucosal fibrosis with dense inflammatory cells, there were no granulomas, which are instead a feature of the CD.

CD free perforation is a rare event and its incidence has been reported to be 1–2 % in Western countries (Ikeuchi and Yamamura 2002; Greenstein et al. 1985, 1987; Abascal et al. 1982). Most perforations in CD occur in the terminal ileum proximal to the stenotic lesion and according to some authors’ opinion from the past decades, used to be an absolute indication for laparotomy (Greenstein et al. 1987; Parray et al. 2011).

Regarding the type of surgical treatment strategies and techniques, the simple suture of the perforation is usually not recommended, because of the high complication and leak rates, the resection of the diseased bowel is suggested instead. In case of small-intestinal perforations, many surgeons agree that limited resection of the most severely affected segment of the intestine, with primary anastomosis, should be performed (Ikeuchi and Yamamura 2002; Greenstein et al. 1985).

The technique for intestinal anastomoses in CD is still controversial, and, to date, there is no evidence of outcome difference between hand-sewn and stapled anastomosis (Scarpa et al. 2003). A temporary end-ileostomy or a diverting loop-ileostomy proximal to the anastomosis, can be considered in CD, especially in case of peritonitis with hemodynamic instability and when the patient’s general conditions and nutritional status are extremely poor (Ikeuchi and Yamamura 2002; Nordentoft et al. 2000; Kesici et al. 2011; Collet e Silva et al. 2000; Neudecker et al. 2002; Agresta et al. 2012; O’Sullivan et al. 1996 Apr; Favuzza et al. 2009; Karoui et al. 2009; Myers et al. 2008; Thomson and Tam 1994; Croft et al. 1995; Smyth et al. 1994; FitzSimmons et al. 1997; Greenstein et al. 1985, 1987; Abascal et al. 1982; Parray et al. 2011).

As already highlighted, laparoscopy has gained wide acceptance in gastrointestinal surgery because of its well-known advantages (Duepree and Senagore 2002; Milson and Hammerhofer 2001; Dunker and Stiggelbout 1998). However, as far as CD is concerned, its application is still on debate. Some authors argue both about missing occult segments of disease and critical proximal strictures due to limited tactile ability,and about the technical difficulty due to fragile inflamed bowel and mesentery and the presence of adhesions, fistulas, and abscesses (Navez et al. 1998). The recent systematic review of RCT’s from Dasari et al. did not find any statistical difference between laparoscopic and open surgery in small bowel CD (Dasari et al. 2011). However, Crohn’s patients are typically young and benefit from a laparoscopic procedure that reduces scar and adhesion formation. In addition, given their high risk of surgical recurrence, CD’s patients benefit from surgical approaches that preserve abdominal wall integrity (Lim et al. 2014; Nguyen et al. 2009). There have been three meta-analyses in the literature on this topic till now (Rosman et al. 2005; Tilney and Constantinides 2006; Tan and Tjandra 2007). All of them agree stating that the laparoscopic group has longer operative times, but faster recovery of bowel function and shorter length of stay. Two of them have found lower morbidity for laparoscopy (Rosman et al. 2005; Tan and Tjandra 2007) (Tan et al. reported 12.8 vs. 20.2 %) and the other one found no difference of complication rate between the two techniques (Tilney and Constantinides 2006). The disease recurrence seems to be similar in the two groups (Stocchi et al. 2008; Lowney et al. 2006) and there does not seem to be statistical significance in the rate of wound infection, anastomotic leak, intra-abdominal abscess, deep vein thrombosis, pneumonia and urinary tract infection (Aarons 2013; Tan and Tjandra 2007).

More recently, the data reviewed from the National Surgical Quality Improvement Program from 2005–2009, on multivariate analysis, show that the laparoscopic ileo-colic resection for CD is a safer choice than the open technique, with fewer complications and shorter hospitalization (Lee and Fleming 2012).

In a recent data evaluation of the Cleveland Clinic Foundation, performed in order to investigate the safety of laparoscopic colorectal surgery as reflected by the anastomotic bowel leak (ABL) rate compared with open surgery, there was no significant statistical difference in the overall clinical ABL rates between laparoscopic and open procedures (2.6% vs2.1 %, *P* = 0.5) (Kessler et al. 2011; El-Gazzaz et al. 2010b).

Analyzing the anastomotic technique issues, along with the refining of the surgical laparoscopic skills and surgeons' increased confidence, the operative technical steps that earlier used to be performed extracorporeally are currently more often being fashioned intracorporeally. In the past, according to Uddo et al., the absolute indication to the intracorporeal anastomosis was the impossibility of performing an extracorporeal anastomosis, like in obese patients with a short mesentery bowel segment (Uddo and Ballantyne 1996).

In case of a right hemi colectomy, since usually the proximal transverse colon hardly can be exteriorized through the umbilical incision, an extracorporeal ileo-colic anastomosis needs a right sub-costal incision to be fashioned. This type of incision is usually associated with a greater postoperative pain and higher risk of surgical site infection, when compared to a fully laparoscopic right colectomy with intracorporeal anastomosis and umbilical extraction of the resected specimen.

Nowadays, the literature has shown that total laparoscopic intracorporeal anastomosis is cost-effective and can be reproducible in experienced hands, without differences in the complication rate if compared to laparoscopic extracorporeal anastomosis. The advantages and disadvantages of the intracorporeal anastomosis compared to the extracorporeal anastomosis are summarized in Table 1 (Bergamaschi et al. 2009; Cirocchi et al. 2013; Chang et al. 2013; Grams et al. 2010; a and Stein and Bergamaschi 2013).

**Table 1:** Advantages and disadvantages of the intracorporeal anastomosis

Advantages	Disadvantages
Less mobilization of the colon	Need of high skilled and trained in laparoscopic suturing surgeons
No manipulation of abdominal organs, in order to reduce adhesions	Higher direct costs of intracorporeal instruments especially the stapler (if compared with extracorporeal)
Shorter extraction site laparotomy, with clinical benefits (less pain and lower rates of wound infection)	Longer operative time (compared with extracorporeal anastomosis) with increased indirect cost and potential higher complication rates
Reduced risk of unrecognized twisting of the terminal ileum mesentery, because of laparoscopic better view	Higher rate of local infections due to the peritoneal contamination of intra-abdominal entero/colotomy with spillage

Considering the clinical and radiological data, in the case-report we described, a perforated diverticulitis (Hinchey IV) was pre-operatively suspected and, according to the 2012 EAES guidelines, the laparoscopic approach could be indicated, depending on the skill of the operator and the clinical stability of the patient, even if the evidence is still too weak for a specific recommendation (LE 3b) (Agresta et al. 2012).

The choice of performing a diagnostic laparoscopy allowed making the correct diagnosis without any other pre-operative procedures and avoiding a dangerous delay in treatment.

Once the diagnosis of faecal peritonitis due to small bowel perforation in a severe ileitis was laparoscopically accomplished, even if the small bowel non-traumatic perforation is not mentioned in the most recent EAES guidelines indications to laparoscopy, we decided to continue with therapeutic laparoscopy and perform the ileo-colic resection, the intracorporeal stapled anastomosis and the abdominal toilette.

The whole surgery was performed by the consultant surgeon (SDS) who has completed a proper laparoscopic training and achieved advanced colorectal laparoscopic skills. An intracorporeal anastomosis, in presence of appropriate skills, might be even easier to perform rather than extracorporeal because of several factors: better view and greater magnification with laparoscopy, the bowel ends to be anastomosed may have less tension and do not need to be stretched and pulled out from the incision (tension is a well known risk factor of dehiscence for the bowel anastomoses) and finally the lesser manipulation of the bowel is reported in the literature to be associated with less trauma to the bowel wall and subsequent better postoperative healing and quicker restoration of peristalsis and bowel function.

In our opinion, the enteric spillage occurred at entero-colotomy definitely did not increase the rate of local infection, since a faecal peritonitis was already there and a proper, careful and complete laparoscopic lavage and toilette has been performed.

The long recovery of the patient with septic shock, wound infection, intra-abdominal inter-loop and pelvic purulent abscesses occurred probably because of the diffuse faecal peritonitis, which can not be precisely dated. All these complications were promptly and effectively resolved with a multidisciplinary approach and no more surgical interventions were needed. Most probably, if underwent a large median laparotomy and the consequent surgical trauma, this patient would have had a much worse postoperative pain and respiratory function requiring both more painkillers and a much longer ventilation, finally most probably leading to development of much more serious complications, such as pneumonia, (e.g. ventilator-associated pneumonia), large pleural effusion (probably requiring chest drain), up to respiratory failure and death.This patient would also definitely have much higher risk of surgical site infections and potential incision/fascial dehiscence or eventration. Laparoscopy and the avoidance of a laparotomy, have instead allowed have instead allowed a potentially more benign postoperative course in an otherwise critically ill patient.

Significant factors influencing the patient’ outcome were the early enteral feeding and the early return of bowel function (POD#4), the absence of anastomotic leak. All of them led to a favourable postoperative course of the patient.

## Conclusions

Although operative times are often longer than open surgery, in presence of experienced operators and selected stable patients, laparoscopic management seems to be a feasible, safe and effective surgical option even in case of faecal peritonitis. In fact, it is proved that the minimally-invasive approach allows high diagnostic and therapeutic accuracy, good short- and long-term outcomes and a faster postoperative recovery. The advantages of minimally invasive surgery in septic patients and patients with peritonitis, are based on less inflammatory response3 with decreased release of pro-inflammatory elements from the mytochondria, ultimately leading to lesser risk of kidney and lung failure (Manfredi and Rovere-querini 2010; Balogh et al. 2012). Intracorporeal anastomoses have been demonstrated to be technically feasible and they may allow better clinical results with lesser need of end stomas (Salomone Di Saverio et al. 2015).

Several studies and meta-analyses have demonstrated the safety and benefits of laparoscopic approach, also for CD surgical treatment. The literature has shown that intracorporeal anastomosis has many advantages, is cost effective and associated with potentially lower complication rates as reported for laparoscopic extracorporeal anastomosis.

To the best of our knowledge, this is the first ever reported case of emergency fully laparoscopic treatment with primary intracorporeal anastomosis in a CD ileitis acute small bowel perforation with faecal peritonitis.

In conclusion, laparoscopic resection and primary intracorporeal anastomosis, maybe be considered a feasible approach to be attempted in patients with perforated CD, even in presence of faecal peritonitis and active inflammatory bowel disease, provided a careful selection of patients, with hemodynamic stability and satisfactory general and nutritional status, when appropriate emergency and colorectal laparoscopic surgical skills are available.

## Additional file

10.1186/s40064-015-1619-x Operative video showing the surgical procedure and technique (operating Surgeon Dr. Salomone Di Saverio MD FACS FRCS)

## References

[CR1] Aarons CB (2013). Laparoscopic Surgery for Crohn Disease : a Brief Review of the Literature. Clin Colon Rectal Surg.

[CR2] Abascal J, Diaz-Rojas F, Jorge J, Sanchez-Vegazo I, Escartin P, Abreu LCC (1982). Free perforation of the small bowel in Crohn’s disease. World J Surg.

[CR3] Agresta F, De Simone P, Bedin N (2004). The laparoscopic approach in abdominal emergencies : a single-center 10-year experience. JSLS.

[CR4] Agresta F, Ciardo LF, Mazzarolo G (2006). Peritonitis: laparoscopic approach. World J Emerg Surg..

[CR5] Agresta F, Ansaloni L, Baiocchi GL (2012). Laparoscopic approach to acute abdomen from the Consensus Development Conference of the Società Italiana di Chirurgia Endoscopica e nuove tecnologie (SICE), Associazione Chirurghi Ospedalieri Italiani (ACOI), Società Italiana di Chirurgia (SIC). Società I. Surg Endosc.

[CR6] Balogh ZJ, Reumann MK, Gruen RL (2012). Advances and future directions for management of trauma patients with musculoskeletal injuries. Lancet.

[CR7] Barbaros U, Ozarmagan S, Erbil Y (2004). Effects of pneumoperitoneum created through CO2 insufflation and parameters of mechanical ventilation (PEEP application) on systemic dissemination of intraabdominal infections. Surg Endosc Other Interv Tech.

[CR8] Bergamaschi R, Haughn C, Reed JF, Arnaud J-P (2009). Laparoscopic intracorporeal ileocolic resection for Crohn’s disease: is it safe?. Dis Colon Rectum.

[CR9] Branicki F. Abdominal emergencies: diagnostic and therapeutic laparoscopy. 2002.http://www.ncbi.nlm.nih.gov/pubmed/?term=BranickiFJ 10.1089/109629602761624234 12542928

[CR10] Casaroli AA, Mimica LMJ, Fontes B, Rasslan S (2011). The effects of pneumoperitoneum and controlled ventilation on peritoneal lymphatic bacterial clearance: experimental results in rats..

[CR11] Casillas S, Delaney CP (2005). Laparoscopic surgery for inflammatory bowel disease. Dig Surg..

[CR12] Chang K, Fakhoury M, Barnajian M. Laparoscopic right colon resection with intracorporeal anastomosis. Surg Endos. 2013. http://www.ncbi.nlm.nih.gov/pubmed/23242489. Accessed 22 Apr 2015 10.1007/s00464-012-2665-x 23242489

[CR13] Chatu S, Poullis A, Holmes R, Greenhalgh R, Pollok RC (2013). Temporal trends in imaging and associated radiation exposure in inflammatory bowel disease. Int J Clin Pract.

[CR14] Cirocchi R, Trastulli S, Farinella E (2013). Intracorporeal versus extracorporeal anastomosis during laparoscopic right hemicolectomy—systematic review and meta-analysis. Surg Oncol.

[CR15] Collet e Silva FD, Ramos RC, Zantut LF, Poggetti RS, Fontes B, Birolini D (2000). Laparoscopic pneumoperitoneum in acute peritonitis does not increase bacteremia or aggravate metabolic or hemodynamic disturbances. Surg Laparosc Endosc Percutan Tech.

[CR16] Croft NM, Marshall TG, Ferguson A (1995). Gut inflammation in children with cystic fibrosis on high-dose enzyme supplements. Lancet.

[CR17] Cueto J. The efficacy of laparoscopic surgery in the diagnosis and treatment of peritonitis. Experience with 107 cases in Mexico City. Surg Endosc. 1997. 11(4): 366–70 10.1007/s004649900365 9094279

[CR18] Dasari BV, McKay D, Gardiner K (2011). Laparoscopic versus open surgery for small bowel Crohn’s disease. Cochrane Database Syst Rev.

[CR19] Di Saverio S (2014). Emergency laparoscopy: a new emerging discipline for treating abdominal emergencies attempting to minimize costs and invasiveness and maximize outcomes and patients’ comfort. J Trauma Acute Care Surg..

[CR20] Duepree H, Senagore A. Advantages of laparoscopic resection for ileocecal Crohn’s disease. Dis colon rectum. 2002. http://link.springer.com/article/10.1007/s10350-004-6253-6. Accessed 22 Apr 2015 10.1007/s10350-004-6253-6 12004208

[CR21] Dunker M, Stiggelbout A. Cosmesis and body image after laparoscopic-assisted and open ileocolic resection for Crohn’s disease. Surg Endosc. 1998. http://link.springer.com/article/10.1007/s004649900851. Accessed 22 Apr 2015 10.1007/s004649900851 9788857

[CR22] El-Gazzaz G, Geisler D, Hull T. Risk of clinical leak after laparoscopic versus open bowel anastomosis. Surg Endosc. 2010. http://link.springer.com/article/10.1007/s00464-009-0867-7. Accessed 23 Apr 2015 10.1007/s00464-009-0867-7 20112117

[CR23] El-Gazzaz G, Geisler D, Hull T (2010). Risk of clinical leak after laparoscopic versus open bowel anastomosis. Surg Endosc.

[CR24] Favuzza J, Friel JC, Kelly JJ, Perugini R, Counihan TC (2009). Benefits of laparoscopic peritoneal lavage for complicated sigmoid diverticulitis. Int J Colorectal Dis.

[CR25] Fazl Q. Parray, Mohd Lateef Wani, Akram H. Bijli, Natasha Thakur, Ifat Irshad and N-H. Crohn’s Disease: A Surgeon’s Perspective. 2011. http://www.ncbi.nlm.nih.gov/pmc/articles/PMC3099084/10.4103/1319-3767.74430 PMC3099084 21196646

[CR26] FitzSimmons SC, Burkhart GA, Borowitz D, Grand RJ, Hammerstrom T, Durie PR, Lloyd-Still JD, Lowenfels AB (1997). High-dose pancreatic-enzyme supplements and fibrosingcolonopathy in children with cystic fibrosis. N Engl J Med.

[CR27] Goyer P, Alves A, Bretagnol F, Bouhnik Y, Valleur P PY. Impact of complex Crohn’s disease on the outcome of laparoscopic ileocecal resection: a comparative clinical study in 124 patients. 2009. http://www.ncbi.nlm.nih.gov/pubmed/?term=Impact+of+complex+Crohn%27s+disease+on+the+outcome+of+laparoscopic+ileocecal+resection%3A+a+comparative+clinical+study+in+124+patients 10.1007/DCR.0b013e31819c9c08 19279413

[CR28] Grams J, Tong W, Greenstein AJ, Salky B (2010). Comparison of intracorporeal versus extracorporeal anastomosis in laparoscopic-assisted hemicolectomy. Surg Endosc.

[CR29] Greenstein AJ, Mann D, Sachar DB AAJ. Free perforation in Crohn’s disease: I. A survey of 99 cases. 1985. http://www.ncbi.nlm.nih.gov/pubmed/3898819 3898819

[CR30] Greenstein AJ, Sachar DB, Mann D, Lachman P, Heimann T, Aufses AH (1987). Spontaneous free perforation and perforated abscess in 30 patients with Crohn’s disease. Ann Surg.

[CR31] Hanly EJ, Mendoza-Sagaon M, Murata K, Hardacre JM, De Maio A, Talamini MA (2003). CO2 Pneumoperitoneum modifies the inflammatory response to sepsis. Ann Surg.

[CR32] Horattas MC, Haller N, Ricchiutti D (2003). Increased transperitoneal bacterial translocation in laparoscopic surgery: relative effects of type of gas and insufflation pressure in an animal model of peritonitis. Surg Endosc Other Interv Tech..

[CR33] Hsieh CS, Tain YL, Chen YC, Chang K, Jean YH, Huang LT (2011). Carbon dioxide pneumoperitoneum induces anti-inflammatory response and hepatic oxidative stress in young rats with bacterial peritonitis. Pediatr Surg Int.

[CR34] Huilgol R. Laparoscopic versus open ileocolic resection for Crohn’s disease. J Laparoendosc Adv Surg Tech A. 2004. http://online.liebertpub.com/doi/abs/10.1089/109264204322973808. Accessed 22 Apr 2015 10.1089/109264204322973808 15107212

[CR35] Ikeuchi H, Yamamura T (2002). Free perforation in Crohn’s disease : review of the Japanese literature. J Gastroenterol.

[CR36] Karoui M, Champault A, Pautrat K, Valleur P, Cherqui D, Champault G (2009). Laparoscopic peritoneal lavage or primary anastomosis with defunctioning stoma for Hinchey 3 complicated diverticulitis: results of a comparative study. Dis Colon Rectum.

[CR37] Kesici U, Kesici S, Polat E (2011). Effects of intra-abdominal pressure increase on intestinal ischemia and bacterial translocation in experimental sepsis model. Saudi Med J.

[CR38] Kessler H, Mudter J, Hohenberger W (2011). Recent results of laparoscopic surgery in inflammatory bowel disease. World J Gastroenterol.

[CR39] Kirshtein B, Roy-Shapira A, Lantsberg L, Mandel S, Avinoach E, Mizrahi S (2003). The use of laparoscopy in abdominal emergencies. Surg Endosc.

[CR40] Lee Y, Fleming FJ, Deeb A-P, Gunzler D, Messing S, Monson JRT (2012). A laparoscopic approach reduces short-term complications and length of stay following ileocolic resection in Crohn’s disease: an analysis of outcomes from the NSQIP database. Colorectal Dis.

[CR41] Lesperance K, Martin MJ, Lehmann R, Brounts L, Steele SR (2009). National trends and outcomes for the surgical therapy of ileocolonic Crohn’s disease: a population-based analysis of laparoscopic vs. open approaches. J Gastrointest Surg.

[CR42] Lim JY, Kim J, Nguyen SQ (2014). Laparoscopic surgery in the management of Crohn’s disease. World J Gastrointest Pathophysiol.

[CR43] Lowney J, Dietz D, Birnbaum E. Is there any difference in recurrence rates in laparoscopic ileocolic resection for Crohn’s disease compared with conventional surgery? A long-term, follow-up study. Dis colon Rectum. 2006. http://link.springer.com/article/10.1007/s10350-005-0214-6. Accessed 22 Apr 2015 10.1007/s10350-005-0214-6 16328612

[CR44] Maartense S, Dunker MS, Slors JFM (2006). Laparoscopic-assisted versus open ileocolic resection for Crohn’s disease: a randomized trial. Ann Surg.

[CR45] Maggiori L, Panis Y (2014). Laparoscopy in Crohn’s disease. Best Pract Res Clin Gastroenterol.

[CR46] Manfredi AA, Rovere-querini P (2010). Clinical implications of basic research the mitochondrion—A trojan horse that kicks off inflammation?. N Engl J Med.

[CR47] Metzelder M, Kuebler JF, Shimotakahara A, Chang DH, Vieten C, Ure B (2008). CO2 Pneumoperitoneum increases survival in mice with polymicrobial peritonitis. Eur J Pediatr Surg.

[CR48] Milson J, Hammerhofer K. Prospective, randomized trial comparing laparoscopic vs. conventional surgery for refractory ileocolic Crohn’s disease. Dis colon Rectum. 2001. http://link.springer.com/article/10.1007/BF02234810. Accessed 22 Apr 2015 10.1007/BF02234810 11805557

[CR49] Montalto AS, Bitto A, Irrera N (2012). CO2 pneumoperitoneum impact on early liver and lung cytokine expression in a rat model of abdominal sepsis. Surg Endosc.

[CR50] Msika S, Iannelli A, Deroide G. Can laparoscopy reduce hospital stay in the treatment of Crohn’s disease?. Dis colon Rectum. 2001. http://link.springer.com/article/10.1007/BF02234387. Accessed 22 Apr 2015 10.1007/BF02234387 11711739

[CR51] Myers E, Hurley M, O’Sullivan GC, Kavanagh D, Wilson I, Winter DC (2008). Laparoscopic peritoneal lavage for generalized peritonitis due to perforated diverticulitis. Br J Surg.

[CR71] Navez B, Tassetti V, Scohy JJ, Mutter D, Guiot P, Evrard S, Marescaux J.(1998) Laparoscopic management of acute peritonitis. Br J Surg 85(1):32–36 10.1046/j.1365-2168.1998.00531.x 9462379

[CR52] Neudecker J, Sauerland S, Neugebauer E (2002). The European Association for Endoscopic Surgery clinical practice guideline on the pneumoperitoneum for laparoscopic surgery. Surg Endosc.

[CR53] Neumann P, Rijcken E, Bruewer M. Current status of laparoscopic surgery for patients with Crohn’s disease. Int J Colorectal Dis. 2013. http://link.springer.com/article/10.1007/s00384-013-1684-y. Accessed 22 Apr 2015 10.1007/s00384-013-1684-y 23588872

[CR54] Nguyen S, Teitelbaum E, Sabnis A. Laparoscopic resection for Crohn’s disease: an experience with 335 cases. Surg Endosc. 2009. http://link.springer.com/article/10.1007/s00464-009-0362-1. Accessed 22 Apr 2015 10.1007/s00464-009-0362-1 19263141

[CR55] Nordentoft T, Bringstrup FA, Bremmelgaard A, Stage JG (2000). Effect of laparoscopy on bacteremia in acute appendicitis: a randomized controlled study. Surg Laparosc Endosc Percutan Tech.

[CR56] O’Sullivan GC, Murphy D, O’Brien MG, Ireland A (1996). Laparoscopic management of generalizedperitonitis due to perforatedcolonicdiverticula. Am J Surg.

[CR57] Patel SV, Patel SVB, Ramagopalan SV, Ott MC (2013). Laparoscopic surgery for Crohn’s disease: a meta-analysis of perioperative complications and long term outcomes compared with open surgery. BMC Surg.

[CR58] Pitombo MB, de Faria CA, Bernardo LC, Steinbruck K, Bernardo Filho M (2008). Dissemination of bacteria labeled with technetium-99 m after laparotomy and abdominal insufflation with different CO2 pressures on rats. Acta Cir Bras.

[CR59] Rosman A, Melis M, Fichera A. Metaanalysis of trials comparing laparoscopic and open surgery for Crohn’s disease. Surg Endosc. 2005. http://link.springer.com/article/10.1007/s00464-005-0114-9. Accessed 23 Apr 2015 10.1007/s00464-005-0114-9 16235128

[CR60] Di Saverio S et al. Techniques and sites of laparoscopic intracorporeal anastomosis in emergency colorectal surgery: how to do it https://www.facs.org/find-a-session/session/10989. Accessed 22 Oct 2015

[CR61] Sangrasi AK, Talpur KAH, Kella N. Role of laparoscopy in peritonitis. 2013;29(4) PMC3817755 24353681

[CR62] Sauerland S, Agresta F, Bergamaschi R (2006). Laparoscopy for abdominal emergencies: evidence-based guidelines of the European Association for endoscopic surgery. Surg Endosc.

[CR63] Scarpa M, Angriman I, Barollo M. Role of stapled and hand-sewn anastomoses in recurrence of Crohn’s disease. Hepatogastroenterology. 2003. http://europepmc.org/abstract/med/15239245. Accessed 22 Apr 2015 15239245

[CR64] Sinha R, Sharma N, Joshi M (2005). Laparoscopic repair of small bowel perforation. JSLS.

[CR65] Smyth RL, vanVelzen D, Smyth AR, Lloyd DA, Heaf DP (1994). Strictures of ascending colon in cystic fibrosis and high-strength pancreatic enzymes. Lancet.

[CR66] Stein SA, Bergamaschi R (2013). Extracorporeal versus intracorporeal ileocolic anastomosis. Tech Coloproctol.

[CR67] Stocchi L, Milsom J, Fazio V. Long-term outcomes of laparoscopic versus open ileocolic resection for Crohn’s disease: follow-up of a prospective randomized trial. Surgery. 2008. http://www.sciencedirect.com/science/article/pii/S0039606008004182. Accessed 22 Apr 2015 10.1016/j.surg.2008.06.016 18847647

[CR68] Tan J, Tjandra J. Laparoscopic surgery for Crohn’s disease: a meta-analysis. Dis Colon Rectum. 2007. http://link.springer.com/article/10.1007/s10350-006-0855-0. Accessed 23 Apr 2015 10.1007/s10350-006-0855-0 17380366

[CR69] Thomson A, Tam P (1994). Case report: colonic stricture and fibrosis associated with high-strength pancreatic enzymes in a child with cystic fibrosis. Br J Radiol.

[CR70] Tilney H, Constantinides V. Comparison of laparoscopic and open ileocecal resection for Crohn’s disease: a metaanalysis. Surg Endosc. 2006. http://link.springer.com/article/10.1007/s00464-005-0500-3. Accessed 22 Apr 2015 10.1007/s00464-005-0500-3 16715212

[CR72] Uddo, J.F.Emicolectomia destra con anastomosi intracorporea. in: G.H. Ballantyne (Ed.) Chirurgia Laparoscopica. Verduci; 1996

[CR73] Uzunkoy A, Ozardali I, Celik H, Demirci M (2012). The effect of carbondioxide pneumoperitoneum on the severity peritonitis. Turkish J Trauma Emerg Surg..

